# Transcriptomic profiling revealed FZD10 as a novel biomarker for nasopharyngeal carcinoma recurrence

**DOI:** 10.3389/fonc.2022.1084713

**Published:** 2023-01-20

**Authors:** Warut Tulalamba, Chawalit Ngernsombat, Noppadol Larbcharoensub, Tavan Janvilisri

**Affiliations:** ^1^ Siriraj Center of Research Excellence in Advanced Gene and Cell Therapy (Si-CORE-AGCT) and Thalassemia Center, Faculty of Medicine Siriraj Hospital, Mahidol University, Bangkok, Thailand; ^2^ Faculty of Science, Mahidol University, Bangkok, Thailand; ^3^ Division of Biochemistry, Department of Preclinical Science, Faculty of Medicine, Thammasat University, Pathumthani, Thailand; ^4^ Department of Pathology, Faculty of Medicine Ramathibodi Hospital, Mahidol University, Bangkok, Thailand; ^5^ Department of Biochemistry, Faculty of Science, Mahidol University, Bangkok, Thailand

**Keywords:** nasopharyngeal carcinoma, transcriptome, microarray, biomarkers, recurrence, Wnt signaling

## Abstract

**Background:**

Nasopharyngeal carcinoma (NPC) is a type of cancers that develops in the nasopharynx, the very upper part of the throat behind the nose. NPC is typically diagnosed in later stages of the disease and has a high rate of recurrence due to the location of the tumor growth site. In this study, we compared the gene expression profiles of NPC tissues from patients with and without recurrence to identify potential molecular biomarkers of NPC recurrence.

**Methods:**

Microarrays were used to analyze the expression of genes in 15 NPC tissues taken at the time of diagnosis and at the site of recurrence following therapeutic treatment. Pathway enrichment analysis was used to examine the biological interactions between the major differentially expressed genes. The target identified was then validated using immunohistochemistry on 86 NPC tissue samples.

**Results:**

Our data showed that the Wnt signaling pathway was enhanced in NPC tissues with recurrence. FZD10, a component of the Wnt signaling pathway, was significantly expressed in NPC tissues, and was significantly associated with NPC recurrence.

**Conclusion:**

Our study provides new insights into the pathogenesis of NPC and identifies FZD10 as a potential molecular biomarker for NPC recurrence. FZD10 may be a promising candidate for NPC recurrence and a potential therapeutic target.

## 1 Introduction

Nasopharyngeal carcinoma (NPC) is a type of head and neck cancer that is uncommon in most parts of the world, but is more prevalent in some regions, such as East and Southeast Asia (1–3). The physical examination of the patient is frequently used to identify cervical lymphadenopathy, which serves as the basis for the diagnosis of NPC. It is common practice to conduct radiographic imaging, including magnetic resonance imaging of the primary and nodal areas and computer tomography to check for bone degradation at the base of the skull, which may be visible at first glance (4). However, due to the vague symptoms and the painless, quiet tumor growth at the primary site, NPC is typically detected at a late stage. Additionally, advanced NPC has a higher risk for metastasis than other head and neck squamous cell carcinomas (5). Although radiotherapy is the recommended course of action for NPC, follow-up research has shown that patients with advanced NPC have poor response rates, locoregional recurrences, and distant metastases (6–8). In order to improve patient outcomes for advanced NPC, concurrent chemotherapy and radiotherapy have been implemented; however, responses from patients with recurrent or metastatic NPC have not been very successful (9, 10). Because NPC has such a poor prognosis, it is critical that we understand the molecular mechanisms behind its pathogenesis and progression, identify appropriate biomarkers for early detection, and search for potential treatment targets (11, 12). Unfortunately, relatively little is known about the pathophysiology of NPC, especially the molecular causes of recurrent NPC.

Gene expression profiling has gained a lot of attention in the fields of biomedicine, especially cancer biology, to retrieve molecular biomarkers. Many public databases, such as ArrayExpress and the Gene Expression Omnibus (GEO), have quickly accumulated transcriptomic data. According to reports, between 76% and 96% of diagnostic platforms based on genetic signatures correctly identify a variety of primary and metastatic cancers (13, 14). Recent articles on transcriptome investigations of NPC in various experimental and clinical settings are available (15, 16). However, efficient biomarkers for NPC recurrence are still unknown. Research on the signalling pathways involved in NPC development is still in its early stages compared to other types of cancer. Therefore, it is very intriguing to study their role in the pathogenesis and growth of NPC in the future.

Here, we conducted a transcriptome analysis to assess the expression profiles of NPC histological tissue sections. The differentially expressed genes in the NPC tissues from patients at the time of diagnosis and the point of recurrence, after receiving therapeutic treatments, were identified using microarray technology. Validation of the target protein was performed using immunohistochemistry. Our findings provide new insights to the pathophysiology of NPC, which may lead to identification of biomarkers for NPC recurrence and potential therapeutics.

## 2 Materials and methods

### 2.1 Nasopharyngeal carcinoma tissues

The tissues that were histopathologically confirmed to be NPC were collected from Ramathibodi Hospital. **Table 1** shows the patient characteristics including age, gender, WHO type, and stage. To evaluate the NPC stages, examiners used physical examination, CT, and MRI according to the TNM categorization system of the AJCC staging system. According to the WHO classification, histological grades were assigned. All patients were treated with concurrent chemotherapy and radiation. The patients received radiation to the primary tumor five times per week at a dose of 18 to 20 Gy, for a total of about 70 Gy. During weeks 1, 4, 7, 10, 13, and 16 of radiotherapy, three rounds of chemotherapy (cisplatin 100 mg/m^2^) were administered concurrently.

**Table 1:** Clinicopathological characteristics of nasopharyngeal carcinoma patients for transcriptomic study.

Relapse	Case no.	Age	Gender	WHO type	TNM stage	
Without NPC relapse	1	60	Female	2a	T1N1M0	2B
	2	47	Female	2b	T4N1M0	4A
	3	53	Female	2b	T2N3M0	4B
	4	30	Female	2b	T2N3M0	4B
	5	51	Male	2a	T3N2M0	3
With 1 NPC relapse	6	46	Female	2a	T4N0M0	4A
	7	44	Male	2a	T3N2M0	3
With 2 NPC relapse	8	43	Female	2a	T2N2M0	3
	9	45	Male	2a	T3N2M0	3

The patients underwent chemotherapy consisting of cisplatin (80 mg/m^2^) on day 1 and 5-fluorouracil (1000 mg/m^2^/day) on days 1 to 5 every 4 weeks for three cycles after the concurrent chemoradiotherapy was finished. All patients were followed up every three to six months for at least 5 years. Tissue samples were fixed in formalin overnight at room temperature. Samples were first dehydrated in a series of ethanol (70%, 90%, and 100%, respectively) and then xylene before being heated to 60°C and embedded in paraffin block. The formalin-fixed paraffin-embedded (FFPE) tissue blocks were stored at room temperature. The use of human materials was approved by the research ethics committee of Faculty of Medicine Ramathibodi Hospital, Mahidol University, which waived the need for consents from the donors.

### 2.2 PALM laser-capture microdissection and pressure catapulting

The FFPE tissues were cut into 8 μm tissue sections and placed on the coated microscope slides as samples for LMPC. This was done using a conventional microtome. After deparaffinization in xylene, tissue sections were rehydrated in a series of decreasing ethanol concentrations (100%, 90%, and 70%, respectively). Tissue slides were dried in a sequence of ethanol before being kept in xylene until laser-capture microdissection, after which they were stained with 0.1% cresyl violet in 50% ethanol for histological markers. Each slide was put onto the PALM^®^ laser-capture microdissection system stage (PALM MicroBeam with PALM RoboMover module and RoboSoftware; Carl Zeiss MicroImaging GmbH, Germany) for light microscopy observation of cresyl violet-stained cells. The laser cut around the NPC cells while being focused through a 40X objective lens, and some cells were then launched into the lid of a 0.6 ml microcentrifuge tube. Per sample and tube, 1 to 2 × 10^6^ NPC cells were dissected and collected. The tubes holding the catapulted cells were then briefly centrifuged to move the cells from the lid to the bottom of the tube for RNA extraction.

### 2.3 RNA preparation

Total RNA from micro-dissected-FFPE samples were extracted using RNeasy FFPE kit (Qiagen, Valencia, CA, USA) according to the manufacturer’s instruction. The extracted RNA was then subjected to a 10-minute RNase-free DNase I treatment at 65°C. Using visible and UV/visible spectrophotometers, a ratio of 260/280 nm absorbance was measured to estimate the amount and purity of the total RNA (Thermo Scientific, Wilmington, DE, USA). A total of 25 ng of total RNA was utilized as a template for TransPlex^®^ Complete Whole Transcriptome Amplification Kit, or WTA (Sigma, St. Louis, MO, USA). In the first phase, sample RNA was reverse transcribed with substantially non-self-complementary primers without 3’ bias. The resulting Omniplex^®^ cDNA library was then amplified by PCR using the universal primer to create WTA products. This library is made up of random, overlapping fragments flanked by universal end sequences. WTA products were re-amplified using REPLI-g FFPE kit (Qiagen, Valencia, CA, USA) according to the company’s recommendation. The resultant amplified products were purified using a PCR purification kit after amplification (Qiagen, Valencia, CA, USA). Using visible and UV/visible spectrophotometers, the concentration and purity of purified cDNA were determined (Thermo Scientific, Wilmington, DE, USA).

### 2.4 Gene expression profiling

Each patient’s amplified cDNA was utilized as a template to stain DNA using Cy3. In brief, 3 μg of DNA were sonicated before being combined with 5 μg of random hexamers (Roche, Madison, WI, USA), 200 μM of dATP/dGTP/dTTP, 100 g of dCTP, 60 g of Cy3-dCTP (GE Healthcare, Little Chalfont, UK), and 10 units of Klenow Fragment (exo-) (Fermentas, Madison, WI, USA) and incubated at 37°C for an overnight period. Cy3-labeled DNA was purified with PCR purification Kit (Qiagen, Valencia, CA, USA). By measuring optical density with visible and UV/visible spectrophotometers (Thermo Scientific Wilmington, DE, USA), the quantity of pure Cy3-labeled cDNA was determined, and frequency of incorporation was estimated. For the gene expression profiling, the Human Whole Genome OneArray™ Version 5 was used (Phalanx Biotech Group, Inc., Hsinchu, Taiwan). Each microarray underwent a pre-hybridization phase in 5X SSPE, 0.1% SDS, and 1% BSA for an hour at 42°C before being spun dry. The microarray was blocked using sheared salmon sperm DNA to lessen non-specific binding (Agilent Technologies, Santa Clara, CA, USA). Three micrograms of Cy3-labeled DNA derived from each NPC sample was hybridized to the microarray. Following 18-h hybridization at 42°C with humidity, non-specific binding was washed away by a series of SSC buffer. The microarray slides were dried by centrifugation. The arrays were then scanned using the Agilent G2565CA Microarray Scanner System with Scan Control Software 8.5 (Agilent Technologies, Santa Clara, CA, USA). The images of microarray slide were exported as a TIFF format.

### 2.5 Data analysis

The Cy3-fluorescent intensities were extracted from the generated images by the Genepix Pro software 6.3 (Molecular Devices, Sunnyvale, CA, USA). The Cy3 signal intensity of each spot was corrected by subtracting background signal intensities in the immediate surroundings. The poor spots were flagged manually and eliminated from the analysis. Following the log_2_ transformation, the normalization was achieved using median with absolute deviation and RANK with RANKIT on SPSS, respectively. The data have been deposited in GEO database (accession number GSE62328). To identify the differentially expressed genes, the MultiExperiment Viewer (MeV) version 4.6 in TM4 software suite (Dana-Farber Cancer Institute, Boston, MA, USA) was used. The level of significance for t-tests statistic was set at P < 0.01. The significant gene lists were selected according to their *P* values and fold difference, both up- and down-regulation. The hierarchical clustering was performed on the set of significant genes to cluster similar groups of samples or genes together. Up-regulated and down-regulated genes then were included in gene set enrichment analysis. Web-based functional annotation tools, WebGastalt (17) was used to analyse clusters of related hits such as enrich signalling pathways (KEGG pathway) as well as gene ontology (GO) term of biological process. Gene set enrichment analysis generated multiple clusters of high significance showed a common trend as to function. The global interaction networks of the protein encoded by differentially expressed genes were predicted using STRING database (18).

### 2.6 qRT-PCR

The expression of selected genes including *CTNNB1*, *FZD10*, and *Wnt8b* was validated through qRT-PCR. The cDNA from FFPE samples were used to perform realtime qRT-PCR using qPCRBIO SyGreen Mix (PCR Biosystems, London, United Kingdom) following the kit’s instruction. The primer sequences are CTNNB1-forward; 5’-AGC TTC CAG ACA CGC TAT CAT-3’; CTNNB1-reverse; 5’-CGG TAC AAC GAG CTG TTT CTA C-3’; Fzd10- forward; 5’-GCT CAT GGT GCG TAT CGG G-3’; Fzd10- reverse; 5’-GAG GCG TTC GTA AAA GTA GCA-3’; Wnt8b- forward; 5’-CAC CTG TGT CCT CCA ACT CA-3’, and Wnt8b- reverse; 5’-CTT CAA TAC CAC TCT GGG CA-3’.

### 2.7 Immunohistochemistry

Tissue microarrays containing 86 human NPC and 6 normal nasopharyngeal epithelial tissues were constructed using paraffin blocks from Ramathibodi Hospital as described previously (19). Briefly, 5 μm TMA sections were blocked with bovine serum albumin and incubated with rabbit anti-FZD10 antibody (1:200 dilution; catalog number ab83044, Abcam, USA) at 4°C overnight in a humidified chamber. The IgG1 was used for negative control. The TMA sections were targeted to stain with SignalStain^®^ Boost IHC Detection Reagent (Cell Signaling Technology, Danvers, MA 01923, USA) for 30 min at room temperature, and visualized with 3-amino-9- ethylcarbazole peroxidase substrate containing H_2_O_2_ for 20 min. Immunohistochemical staining was evaluated independently by 2 pathologists. The FZD10 labeling was divided into five grades: 0 (no FZD10 labeling), 1 (>0-25% cells labeling), 2 (26-50% cells labeling), 3 (51-75% cells labeling), and 4 (76-100% cells labeling). The staining intensity for FZD10 were categorized as 0 for negative, 1 for weakly positive, 2 for moderately positive, and 3 for strongly positive. The staining positivity was calculated by the formula: labeling score × intensity score. A score less than 4 was considered low FZD10 expression while score 4-12 indicated high expression. The correlation between expression of FZD10 protein and NPC clinical features was evaluated by Pearson Chi-Square test. All *P*-values were calculated based on two-sided statistical analysis, and a probability level < 0.05 was considered statistically significant.

## 3 Results

### 3.1 Differentially expressed genes involved in NPC recurrence

**Figure 1:**
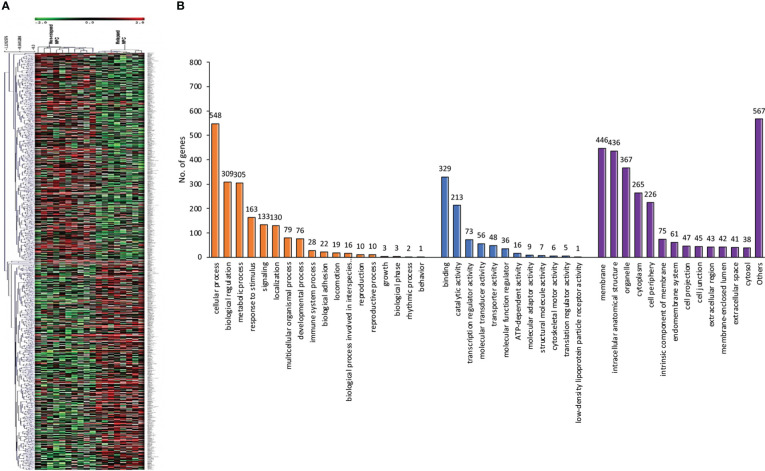
Differentially expressed genes involved in NPC recurrence. **(A)** Hierarchical clustering of significant genes involved in NPC recurrence. Each row represents each significant gene, and each column corresponds to a specific sample. The status of each gene is indicated by the intensity as indicated by the scale bar with log_2_ scale. **(B)** Gene ontology classification of differentially expressed genes. A bar represents each gene ontology category. The height of the bar represents the number of genes observed in each category. The number of genes per category is indicated upon the bars text.

Using a non-parametric approach, the mRNA profiles of NPC tissues at the time of diagnosis from patients with and without relapse were compared. **Table 1** displays the clinicopathological features of our NPC cohort. A total of 1,180 genes showed substantially different expression between NPC with and without disease recurrence following treatment, with a *P*-value of less than 0.05. **Figure 1A** shows a hierarchical clustering (HCL) tree of significant genes. According to HCL, genes with similar expression patterns were clearly clustered together and connected by a network of branches that showed which genes were up- and down-regulated. Top 50 significant up- and down-regulated genes are summarized in **Table S1**. To further shed light on the biological activities of differentially expressed genes in NPC, we carried out a gene ontology analysis to group these genes into clusters based on their biological functions, cellular components, and molecular functions (**Figure 1B**). Our findings showed that more than 50% of the genes were involved in biological activities such as biological regulation, metabolism, and cell signaling, while less than 5% of the genes were involved in growth. Fourteen categories of cellular compartments were identified. Most of the differentially expressed genes were primarily found in the membrane and intracellular compartments. Protein binding, catalytic activity, and transcriptional regulator activity were among the top ranks in molecular function.

### 3.2 Identification of key signaling pathway of recurrent NPC

**Figure 2:**
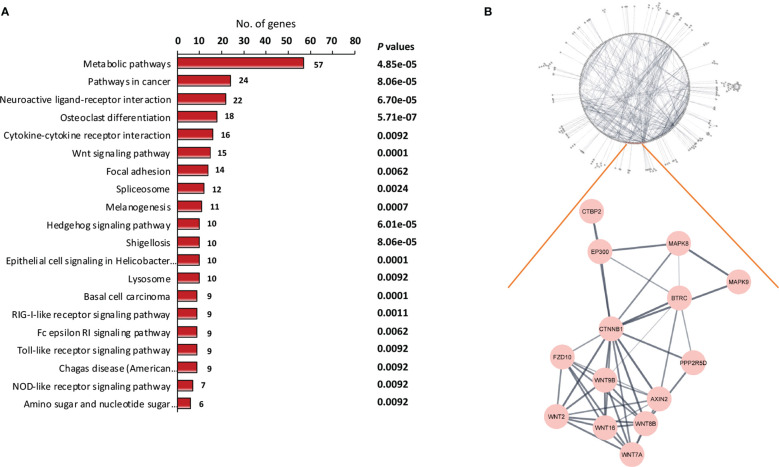
Identification of key signaling pathway of recurrent NPC. **(A)** Pathway enrichment analysis of differentially expressed genes. The differentially expressed genes between relapse and non-relapse NPC were identified using t test with *P* values less than 0.01 and were used as input into pathway enrichment analysis. Based on KEGG database, multiple clusters of highly significant pathways in NPC were identified. **(B)** Protein-protein interaction networks were discovered *via* network analysis. Among the dysregulated mechanisms in NPC recurrence is the Wnt signaling pathway. Several Wnt pathway members have been found to be differentially expressed in recurrent NPC.

KEGG pathway enrichment analysis was carried out to gain an understanding of the functional roles of differentially expressed genes. **Figure 2A** shows 20 enriched KEGG pathways that were retrieved at a significant level of *P* < 0.01. The osteoclast differentiation pathway, RIG-I-like receptor signaling pathway, antiviral innate immunity pathway, and several carcinogenic pathways were among the notable KEGG pathways that we discovered. The carcinogenic pathways, Hedgehog and Wnt, are well known, and have been shown to be implicated in malignancies like NPC in the past. To confirm the importance of the projected molecular pathways from gene enrichment studies in NPC, we built protein-protein networks using STRING from the genes of the top 20 pathways in the KEGG pathway database (**Figure 2B**). In this work, the Wnt signaling pathway was highlighted. In the relapse cases, it was discovered that several Wnt-related genes were dysregulated (**Table 2**).

**Table 2:** List of differentially expressed genes between relapsed and non-relapsed NPC following Wnt signaling pathway enrichment analysis.

Wnt signaling pathway		KEGG ID: 04310	*P* = 0.0001
Index	Symbol	Gene Name	EntrezGene
1	*AXIN2*	axin 2	8313
2	*BTRC*	beta-transducin repeat containing E3 ubiquitin protein ligase	8945
3	*CTBP2*	C-terminal binding protein 2	1488
4	*CTNNB1*	catenin (cadherin-associated protein), beta 1, 88kDa	1499
5	*EP300*	E1A binding protein p300	2033
6	*FZD10*	frizzled family receptor 10	11211
7	*MAPK8*	mitogen-activated protein kinase 8	5599
8	*MAPK9*	mitogen-activated protein kinase 9	5601
9	*PPP2R5D*	protein phosphatase 2, regulatory subunit B’, delta	5528
10	*PRKX*	protein kinase, X-linked	5613
11	*WNT16*	wingless-type MMTV integration site family, member 16	51384
12	*WNT2*	wingless-type MMTV integration site family member 2	7472
13	*WNT7A*	wingless-type MMTV integration site family, member 7A	7476
14	*WNT8B*	wingless-type MMTV integration site family, member 8B	7479
15	*WNT9B*	wingless-type MMTV integration site family, member 9B	7484

We verified the accuracy of our transcriptomic findings using quantitative RT-PCR. With the use of the 2^-ΔΔCt^ method and ACTB as an endogenous reference, the expression of Wnt-related genes was quantified in the cDNA from FFPE samples of non-relapse and relapse NPC patients. β-catenin, FZD10, and WNT8B proteins were selected for validation. The Spearman’s rho test showed that the correlation between the microarray and qPCR results was 0.837 (**Supplementary Figure S1**).

### 3.3 FZD10 as a biomarker for NPC recurrence

**Figure 3:**
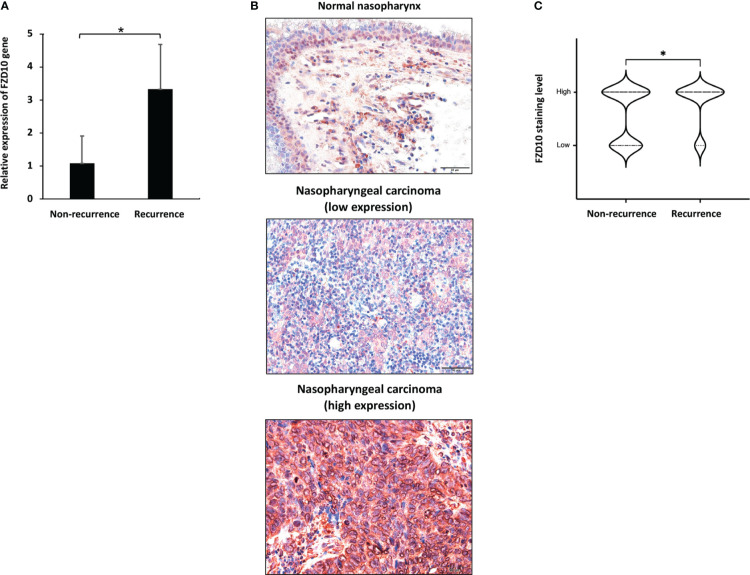
FZD10 as a biomarker for NPC recurrence. **(A)** The relative *FZD10* mRNA level of non-recurrent NPC compared to recurrent NPC. **(B)** FZD10 protein expression in normal nasopharynx and NPC tissues. Shown are representative photomicrographs at magnification X200 of normal nasopharynx and cancerous nasopharyngeal tissues that were subjected to immunostaining of FZD10. **(C)** The correlation of recurrent status of NPC patients and the FZD10 expression level. The NPC tissues was stratified into non-recurrent (N=59) and recurrent (N=27). Recurrent NPC represented higher FZD10 expression than non-recurrent NPC with *P* value of 0.044. *P < 0.05

To further investigate the role of FZD10 in NPC recurrence, we initially conducted qPCR on NPC tissue samples from patients with and without relapse at the time of diagnosis (**Table 1**). Our results showed that the relative expression of the *FZD10* gene was higher in NPC tissues with recurrence compared to those without relapse (**Figure 3A**). We then performed immunohistochemistry on a cohort of 86 NPC and 6 normal nasopharyngeal tissue samples to assess the level of FZD10 protein expression (**Table 3**). The staining patterns for FZD10 protein were mostly found in the cytoplasm of both normal and NPC tissues, as shown in **Figure 3B**. We also evaluated the relationship between FZD10 expression and clinicopathological features in NPC patients. Our findings revealed that elevated FZD10 expression was observed in 57/86 (66.3%) of NPC patients and was significantly associated with recurrence status (*P* = 0.044) (**Figure 3C**). However, FZD10 expression was not significantly associated with age, gender, WHO classification, AJCC staging, T stage, lymph node metastasis, or systemic metastasis (*P* > 0.05).

**Table 3:** Clinicopathological characteristics of nasopharyngeal carcinoma patients in this study.

Characteristics		No. of patients	
**Age**			
	mean	50.3	years
	median	51.0	years
	range	16-77	years
**Gender**			%
	Male	62	72.1
	Female	24	27.9
**WHO classification**			%
	Type 1	2	2.3
	Type 2	51	59.3
	Type 3	33	38.4
**AJCC staging**			%
	Stage I-II	11	12.8
	Stage III-IV	73	84.9
	N/A	2	2.3
**T stage**			%
	T1-T2	31	36.0
	T3-T4	54	62.8
	N/A	1	1.2
**Regional lymph node metastasis**			%
	No	21	24.4
	Yes (N1-N3)	65	75.6
**systemic metastasis**			%
	No	74	86.0
	Yes (M1)	11	12.8
	N/A	1	1.2
**recurrence**			%
	No	59	68.6
	Yes	27	31.4
**FZD10 expression**			%
	Low	29	33.7
	High	57	66.3
	Total	86	100.0

N/A, Not available.

Based on our cohort, there was no statistically significant association between NPC patient survival and FZD10 expression. The data from the human protein atlas (https://www.proteinatlas.org/) of head and neck cancer were then used to analyze patient survival with varied Fzd10 expression levels. The 499 patients with head and neck cancer were divided into two groups: those with low Fzd10 expression (n = 343) and those with high Fzd10 expression (n = 156). The Kaplan-Meier survival analysis revealed that those with low Fzd10 mRNA expression outlived those with high expression (*P* = 0.046) (**Supplementary Figure S2**).

## 4 Discussion

Most NPC patients present in later stages and have a poor prognosis due to metastasis and the recurrence of the disease. However, to date, there is no report on the molecular biomarkers for NPC recurrence as the recurrence often occurs months or years after the primary diagnosis and treatment and obtaining tissue samples from patients at this point is difficult. Moreover, tissue samples from patients with recurrent disease are limited in quantity or quality because of previous treatment. A lack of knowledge of molecular pathophysiology and adequate biomarkers contributes to a poor response to existing therapy. In this study, the NPC biopsies from patients were taken at the time of diagnosis and after relapses following therapy to better understand the pathophysiology of NPC recurrence. The expression patterns of NPCs that had relapsed and those that had not were examined to identify the key players in NPC development and therapeutic response. A total of 1,180 genes were significantly identified. Among the significantly enriched pathways, the osteoclast differentiation pathway is predominant. The MAPK and NF-B gene families, which are present in other important pathways including the focal adhesion pathway and the Fc epsilon RI signaling pathway, are among many relevant genes enriched in this study (20, 21). The signaling pathway of the RIG-I-like receptors, which are important in antiviral innate immunity (22), was also discovered. MAVS (mitochondrial antiviral signaling protein), one of the key components of this signaling, is dysregulated in relapsed NPC. In response to viral infection, MAVS, a membrane-bound protein, transmits signals from RIG-I to downstream signaling molecules *via* the transcription factors NF-B, IRF3, and IRF7 to create inflammatory cytokines such as IFN-beta (23). There is no direct data of MAVS dysregulation in NPC yet. Evidence suggests that EBV infection is linked to recurrent NPC, and plasma EBV DNA is essential for the early identification of local or distant failure (24, 25). Increased MAVS expression in relapsed NPC may indicate the presence of EBV infection, which is causally connected to the etiology and recurrence of NPC. The radiation treatment of NPC can cause microvasculature damage by affecting the tumor microenvironment (26, 27). Hyperactivation of the hedgehog (HH) pathway was also upregulated in relapsed NPC. The basal cell carcinoma related pathway is also enriched, which is closely associated with hedgehog signaling. The HH pathway is dysregulated in head and neck cancer, including NPC. Aberrant of GLI1 has been reported in NPC tissues and cell lines (28). Interestingly, HH signaling has been associated to NPC metastasis, as suggested by the effect of *MTA1* (metastasis-associated gene 1) on the aggressive phenotypes of NPC cells (29). Moreover, the stem cell-like characteristics of NPC are maintained by EBV infection through the HH signaling pathway (30). Overall, our data suggest that a few factors contribute to the recurrence of NPCs, and further research is necessary to confirm their contributions.

The Wnt signaling pathway plays an important role in cancer development. Dysregulation of this pathway can cause unwanted cell growth and movement, which can lead to tumor development, including NPC (2, 31). Additionally, it has been suggested that the Wnt signaling pathway contributes to fibrosis by interacting with other signals, such as TGF- β, and inducing fibroblast activation and fibrogenesis (32). Our transcriptomic analyses resulted in the considerable identification of the Wnt signaling pathway using pathway enrichment analysis. Several Wnt signaling pathway members have previously been identified as being up-regulated in several types of cancers, including NPC (19, 33 – 35). Remarkably, the relapse cases of NPC exhibited the different expression of Wnt signaling compared to the point of diagnosis, including various *WNT* isoforms, *FZD10*, *CTNNB1*, and *AXIN2* expression. Moreover, up-regulation of WNT5A has recently been reported in primary NPC tissue samples and cell lines associated with EBV infection (36), which promotes aggressiveness and stem characteristics in NPC (37). Our previous tissue microarray analysis illustrated that WNT8B is associated with NPC patients’ survival, which could be used as a prognostic biomarker for NPC patients (19). As the NPC development comprises multi-step carcinogenesis, the differential expression of Wnt components in our studies may affect the mechanisms of NPC pathogenesis.

Frizzled (FZD) is a protein that is crucial for many aspects of cell development, including cell polarity, cell proliferation, and the development of both embryonic and adult cells. It is also a potential target for the treatment of cancer (38). Several Frizzled isoforms have been identified as possible targets for treating human cancer. FZD10 is among the genes that are aberrantly expressed in the Wnt signaling pathway. Previous studies have shown that FZD10 is involved in the progression of several types of cancer, including colorectal cancer, gastric cancer, and synovial sarcomas (39 – 41). Additionally, a therapeutic targeting FZD10 (OTSA101) is currently in clinical trials (42). However, the role of FZD10 in NPC has not been thoroughly studied, making it a potential target for further research. The immunohistochemical analyses suggested that high FZD10 expression was significantly associated with recurrent NPC. Based on our validating cohort, there was no correlation between other clinicopathological parameters including survival with FZD10. This is possibly due to the small cohort. As NPC is a distinct subtype of head and neck cancer, we evaluated the mRNA expression of FZD10 in head and neck cancer based on the Human Protein Atlas datasets and found that it is associated with the poor prognosis. It is noteworthy that although there is several mRNA profiling on NPC tissues, the patient characteristics including survival are not readily available in the public databases. In addition, a study reported that FZD10 expression negatively exhibited correlation with overall survival of NPC patients in a Chinese cohort (43). Moreover, FZD10 was upregulated and cross talked with TGF-β1, which activate the HH signaling pathway in myofibroblast differentiation and pulmonary fibrosis (44). To date, there has been no report on the role of FZD10 in the recurrent cancers. Other Frizzled isoforms have been associated with recurrence in some cancers. For example, FZD2 was upregulated in the hepatocellular carcinoma and was significantly associated metastasis and cancer recurrence (45). FZD3 expression was correlated with recurrent or metastatic CRC (46). Triple-negative breast cancer was found to highly express FZD5 mRNA, which correlated with shorter overall survival, recurrence-free survival, distal metastasis-free survival, and post-progression survival (47). Therefore, one of the key signaling pathways involved in the development of NPC was the Wnt signaling pathway. FZD10 may be an important cancer recurrent biomarker and a potentially effective target for cancer therapy.

In summary, our transcriptomic findings identified several signaling pathways involved in NPC recurrence. One of these mechanisms, Wnt signaling, appears to be a disrupted during recurrence and its participation may help is better understand how NPC recurrence is regulated. FZD10, a Wnt signaling receptor, is involved in NPC recurrence, and could serve as an independent predictive biomarker for cancer recurrence. Further research into prospective FZD10-targeted therapies is necessary, as it may improve the survival outcomes of these patients.

## Data availability statement

The data presented in the study are deposited in the Gene Expression Omnibus repository, accession number GSE62328 (https://www.ncbi.nlm.nih.gov/geo/query/acc.cgi?acc=GSE62328/).

## Ethics statement

The studies involving human participants were reviewed and approved by the research ethics committee of Faculty of Medicine Ramathibodi Hospital, Mahidol University. Written informed consent for participation was not required for this study in accordance with the national legislation and the institutional requirements.

## Author contributions

Conceptualization: TJ. Methodology: WT, CN and TJ. Validation: CN. Investigation: WT and CN. Resources: NL and TJ. Writing—original draft preparation: WT and CN. Writing—review and editing: CN and TJ. Supervision: TJ. Project administration: TJ. Funding acquisition: TJ. All authors contributed to the article and approved the submitted version.
