# Supplementary material for: Transcriptomic profiling revealed FZD10 as a novel biomarker for nasopharyngeal carcinoma recurrence
Source: Front Oncol. 2023 Jan 20;12:1084713. doi: 10.3389/fonc.2022.1084713 (PMC9909960; doi:10.3389/fonc.2022.1084713)
Supplement: Supplementary file 1 [file DataSheet_1.pdf]

Supplementary data

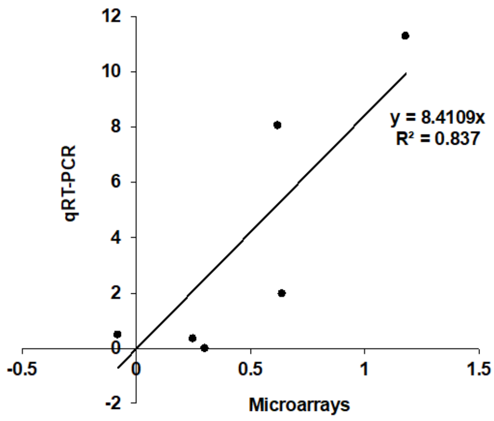

Figure S1 Validation of microarray data by qRT-PCR. Gene expression changes in NPC tissue samples measured by microarray analysis or qRT-PCR are compared.

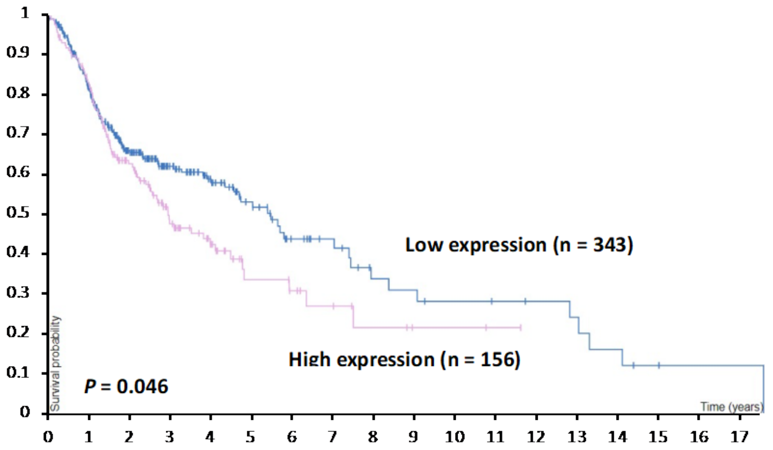

Figure S2 Kaplan-Meier plot from the analysis of correlation between *FZD10* mRNA expression level and head and neck cancer patient survival (Log- rank *P* value = 0.046). The data are retrieved from the public cohort of the Human Protein Atlas v.21.1 (https://www.proteinatlas.org/ENSG00000111432-FZD10/pathology/head+and+neck+cancer).

Table S1 Top 50 differentially genes expressed in relapsed NPC patients.

A. List of top 50 up-regulated genes

Gene	Gene name	*P* value
HP1BP3	heterochromatin protein 1, binding protein 3	0.0008551
PCDHGA9	protocadherin gamma subfamily A, 9	0.0010692
MAVS	mitochondrial antiviral signaling protein	0.0012446
LOC257358	uncharacterized LOC257358	0.0016581
INTS4L1	integrator complex subunit 4-like 1	0.0020756
BTF3	basic transcription factor 3	0.0024839
CASQ1	calsequestrin 1 (fast-twitch, skeletal muscle)	0.0025196
CTXN3	cortexin 3	0.0025549
APCS	amyloid P component, serum	0.0029849
MXD3	MAX dimerization protein 3	0.0033665
HSPC157	Homo sapiens HSPC157 mRNA, complete cds.	0.0034148
SFRS11	serine/arginine-rich splicing factor 11	0.0034148
ATPIF1	ATPase inhibitory factor 1	0.0036038
CHD4	chromodomain helicase DNA binding protein 4	0.0036038
SYDE2	synapse defective 1, Rho GTPase, homolog 2 (C. elegans)	0.0036038
WDR36	WD repeat domain 36	0.0038135
RAPGEF6	Rap guanine nucleotide exchange factor (GEF) 6	0.0038924
CDKAL1	CDK5 regulatory subunit associated protein 1-like 1	0.0042747
HLA-J	major histocompatibility complex, class I, J (pseudogene)	0.0044833
ATPAF1	ATP synthase mitochondrial F1 complex assembly factor 1	0.0045087
SMARCD2	SWI/SNF related, matrix associated, actin dependent regulator of chromatin, subfamily d, member 2	0.0045087

C9orf31	chromosome 9 open reading frame 31	0.0045744
MTA3	metastasis associated 1 family, member 3	0.0045744
PFKM	phosphofructokinase, muscle	0.0048009
KRT16	keratin 16	0.0050308
SPG7	spastic paraplegia 7 (pure and complicated autosomal recessive)	0.0050308
TM6SF2	transmembrane 6 superfamily member 2	0.0050308
HES4	hairy and enhancer of split 4 (Drosophila)	0.0052622
LINGO4	leucine rich repeat and Ig domain containing 4	0.0052622
CSTB	cystatin B (stefin B)	0.0054786
ERI2	ERI1 exoribonuclease family member 2	0.0058796
ITPR3	inositol 1,4,5-trisphosphate receptor, type 3	0.0061699
SLC7A2	solute carrier family 7 (cationic amino acid transporter, y+ system), member 2	0.0061699
SYT14	synaptotagmin XIV	0.0061699
TOP3B	topoisomerase (DNA) III beta	0.0062697
DNMT3L	DNA (cytosine-5-)-methyltransferase 3-like	0.0062852
SSH1	slingshot homolog 1 (Drosophila)	0.0063229
IDH3G	isocitrate dehydrogenase 3 (NAD+) gamma	0.0066419
C1orf68	chromosome 1 open reading frame 68	0.0067166
DQX1	DEAQ box RNA-dependent ATPase 1	0.0067166
FAM57B	family with sequence similarity 57, member B	0.0067166
RRN3P2	RNA polymerase I transcription factor homolog (S. cerevisiae) pseudogene 2	0.0067166
C11orf82	chromosome 11 open reading frame 82	0.0069505

EP400	E1A binding protein p400	0.0069505
FAU	Finkel-Biskis-Reilly murine sarcoma virus (FBR-MuSV) ubiquitously expressed	0.0069505
LYZ	lysozyme	0.0069505
MYST4	K(Lysine) Acetyltransferase 6B	0.0069505
HAUS1	HAUS augmin-like complex, subunit 1	0.0070536
GLT25D1	glycosyltransferase 25 domain containing 1	0.0076858
SERBP1	SERPINE1 mRNA binding protein 1	0.0077740

B. List of top 50 down-regulated genes

**Gene**	**Gene name**	***P* value**
RICH2	Rho GTPase Activating Protein 44	0.0007344
PCDHA11	protocadherin alpha 11	0.0011313
SLC12A6	solute carrier family 12 (potassium/chloride transporters), member 6	0.0011381
PARP14	poly (ADP-ribose) polymerase family, member 14	0.0012798
ALG2	asparagine-linked glycosylation 2, alpha-1,3-mannosyltransferase homolog (S. cerevisiae)	0.0013806
ETHE1	ethylmalonic encephalopathy 1	0.0014627
SPTY2D1	SPT2, Suppressor of Ty, domain containing 1 (S. cerevisiae)	0.0016292
ARF1	ADP-ribosylation factor 1	0.0017909
DUS3L	dihydrouridine synthase 3-like (S. cerevisiae)	0.0018719
SLC48A1	solute carrier family 48 (heme transporter), member 1	0.0025196
ASB3	ankyrin repeat and SOCS box containing 3	0.0026998
C14orf23	chromosome 14 open reading frame 23	0.0032162

HSPBAP1	HSPB (heat shock 27kDa) associated protein 1	0.0032162
CALN1	calneuron 1	0.0033665
ENG	endoglin	0.0033665
CLSTN2	calsyntenin 2	0.0034053
OR4P4	olfactory receptor, family 4, subfamily P, member 4	0.0036038
SLC6A17	solute carrier family 6, member 17	0.0036038
SLC16A7	solute carrier family 16, member 7 (monocarboxylic acid transporter 2)	0.0038135
MAPK9	mitogen-activated protein kinase 9	0.0038924
APOE	apolipoprotein E	0.0042747
TRAF5	TNF receptor-associated factor 5	0.0042747
C12orf57	chromosome 12 open reading frame 57	0.0044833
SLC7A6OS	solute carrier family 7, member 6 opposite strand	0.0044833
POLR2B	polymerase (RNA) II (DNA directed) polypeptide B, 140kDa	0.0045087
SLMAP	sarcolemma associated protein	0.0045087
CHERP	calcium homeostasis endoplasmic reticulum protein	0.0045744
ATP5J	ATP synthase, H+ transporting, mitochondrial Fo complex, subunit F6	0.0046532
C16orf54	chromosome 16 open reading frame 54	0.0046532
RBM34	RNA binding motif protein 34	0.0046532
TMEM63A	transmembrane protein 63A	0.0046532
PCDHB5	protocadherin beta 5	0.0046777
CDK20	cyclin-dependent kinase 20	0.0050308
SNX32	sorting nexin 32	0.0052345
ITGB1BP3	integrin beta 1 binding protein 3	0.0052622

C2orf72	chromosome 2 open reading frame 72	0.0054139
COPS8	COP9 constitutive photomorphogenic homolog subunit 8 (Arabidopsis)	0.0054786
ETF1	eukaryotic translation termination factor 1	0.0054786
SLC34A3	solute carrier family 34 (sodium phosphate), member 3	0.0058796
COQ10B	coenzyme Q10 homolog B (S. cerevisiae)	0.0061699
EVI2A	ecotropic viral integration site 2A	0.0061699
MAPKSP1	mitogen-activated protein kinase scaffold protein 1	0.0061699
PPARGC1B	peroxisome proliferator-activated receptor gamma, coactivator 1 beta	0.0062697
CC2D1B	coiled-coil and C2 domain containing 1B	0.0062852
SAAL1	serum amyloid A-like 1	0.0062852
MGAT4A	mannosyl (alpha-1,3-)-glycoprotein beta-1,4-N-acetylglucosaminyltransferase, isozyme A	0.0063229
PHLDB2	pleckstrin homology-like domain, family B, member 2	0.0063229
CCDC126	coiled-coil domain containing 126	0.0064853
MIXL1	Mix paired-like homeobox	0.0066419
CHRM5	cholinergic receptor, muscarinic 5	0.0066953
